# Surgical Aortic Valve Replacement Following Transcatheter Aortic Valve Implantation Degeneration in a Patient with Prior David Procedure: A Case Report

**DOI:** 10.1055/a-2938-6221

**Published:** 2026-09-02

**Authors:** Theodor Tibeau, Tulio Caldonazo, Rodrigo Saadi, Angelique Runkel, Sultonbek Toshmatov, Murat Mukharyamov, Hristo Kirov, Torsten Doenst

**Affiliations:** 1 Botucatu Medical School, Sao Paulo State University (UNESP)BotucatuBrazil; 2 Department of Cardiothoracic SurgeryJena University Hospital, Friedrich Schiller University of JenaJenaGermany; 3 Department of Cardiovascular Surgery, Hospital Mãe de DeusPorto AlegreBrazil

**Keywords:** aortic valve insufficiency, TAVI failure, surgical aortic valve replacement, David procedure

## Abstract

This is the first reported case of a self-expanding aortic valve explanted from the ascending aorta consisting of a Dacron prosthesis. We describe a 75-year-old male with bicuspid aortic valve regurgitation initially treated with a David procedure. Following degeneration, a “valve-in-repaired-valve” transcatheter aortic valve implantation (TAVI) was performed. Six years later, he presented with dyspnea, severe insufficiency, and paravalvular leakage. An interdisciplinary team opted for redo surgery. The patient underwent successful redo aortic valve replacement with an EpicMax bioprosthesis via resternotomy. Despite a complex postoperative course, he recovered well. This case underscores key challenges in lifetime management, including TAVI durability, sizing, and reoperative strategies.

## Introduction

Aortic valve insufficiency following transcatheter aortic valve implantation (TAVI) is a challenging complication, especially in patients with prior cardiac surgery. 1 2 In patients with a history of prior complex aortic root surgery such as the David procedure, therapeutic options must be tailored individually. Redo sternotomy for surgical aortic valve replacement (SAVR) in the setting of a failed TAVI poses significant technical demands, as demonstrated by the EXPLANT-TAVR registry, which reported considerable perioperative risk and frequent need for concomitant procedures. 3 Long-term durability of valve-sparing root replacement has demonstrated favorable outcomes, with 90% freedom from reoperation at 10 to 15 years. 4 5 However, long-term durability of TAVI valves remains less well established, particularly in patients with bicuspid aortic valves, further complicating clinical decision-making in this setting. 6 Here, we present a case of successful SAVR in a 75-year-old man with prior David procedure, that lasted 13 years before TAVI and who subsequently developed progressive aortic valve insufficiency. To our knowledge, this is the first reported case of surgical explantation of a failed TAVI with subsequent implantation of a surgical bioprosthesis in a patient with a prior valve-sparing aortic root replacement.

## Case Description

A 75-year-old man presented to our outpatient clinic for evaluation of newly diagnosed prosthetic aortic valve insufficiency. His cardiac history included bicuspid aortic valve reconstruction with a composite graft (David procedure) in 2005, and TAVI with a 34-mm CoreValve Evolut R prosthesis in 2018 due to severe aortic stenosis of his native bicuspid valve. The TAVI procedure was uneventful and initially successful, with good prosthesis function during follow-up.

In late 2024, transthoracic and transesophageal echocardiography revealed moderate to severe aortic valve insufficiency due to degeneration of the TAVI prosthesis. The patient reported progression of chest pressure at rest and on exertion and dyspnea. Blood pressure measurements consistently showed systolic hypertension with low diastolic values. NT-proBNP was elevated at 736 pg/mL. Left ventricular function was preserved. Given these findings, and after multidisciplinary Heart Team discussion, a surgical approach with TAVI explant and implantation of a surgical bioprosthesis was indicated.

The patient underwent elective redo median sternotomy under general anesthesia. Significant adhesions were encountered but were mostly easily detached with careful dissection. The pericardium was found to be mostly closed. Palpation revealed a normal aorta, and the old graft (David prosthesis) was well visible, widely adherent to the surrounding tissue. Arterial cannulation was performed in the ascending aorta, and venous cannulation was established through the femoral vein.

Cardiopulmonary bypass (CPB) was initiated and cardioplegia was administered using 1,900 mL of Bretschneider solution, antegradely into the aortic root to cool the TAVI prosthesis and retrogradely via the coronary sinus. Complete cardiac arrest was achieved approximately 20 seconds after cardioplegia infusion.

**Fig. 1:**
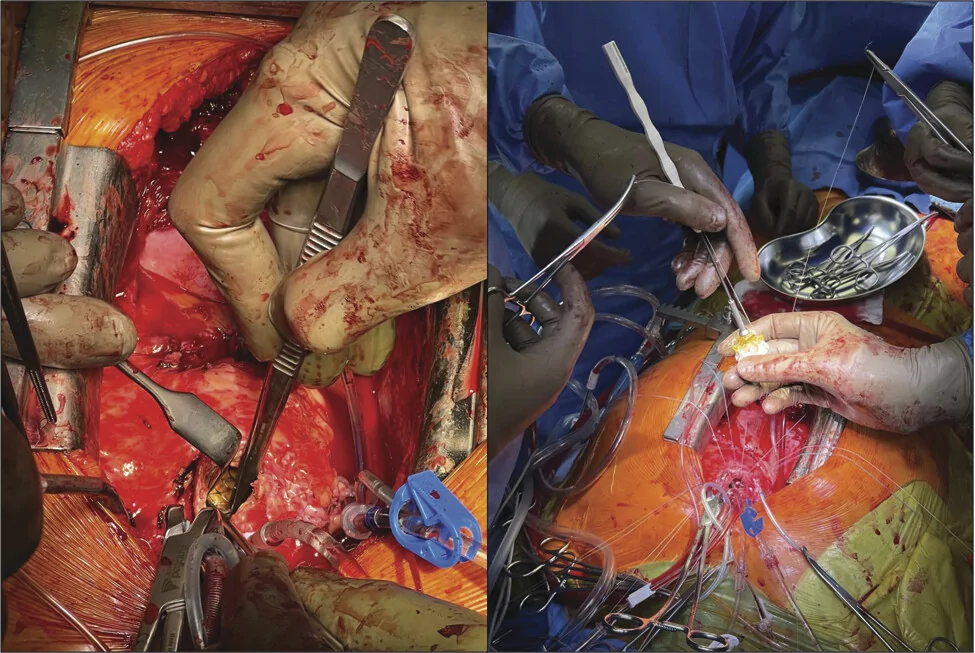
Intraoperative figures showing the surgical exposure and valve replacement.

**Fig. 2:**
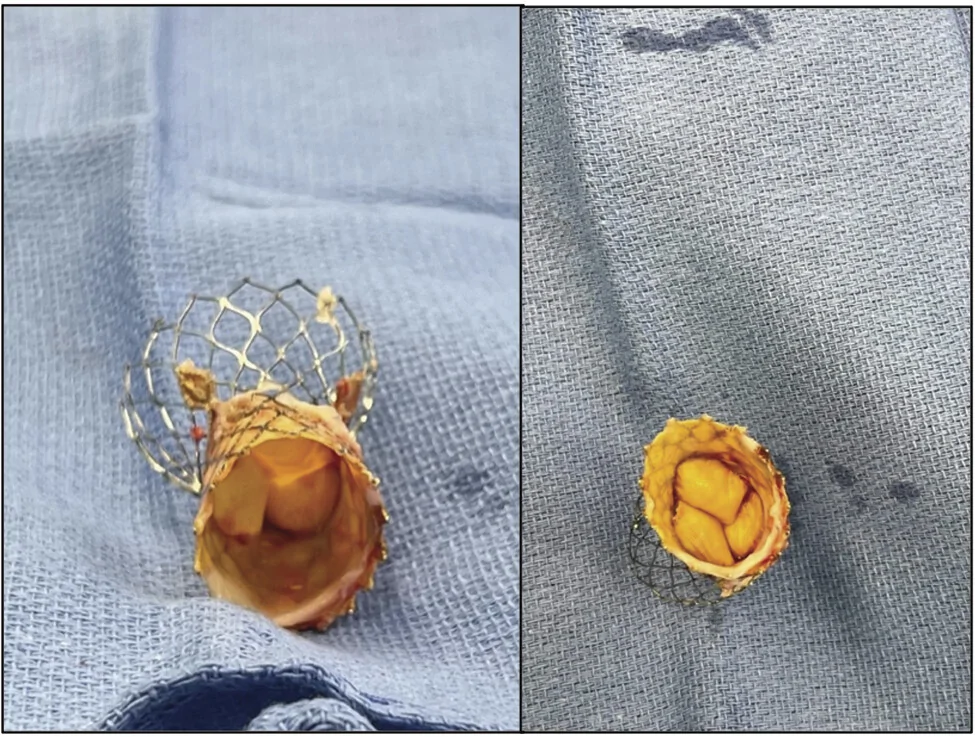
TAVI explanted without significant deformities. TAVI, transcatheter aortic valve implantation.

A transverse aortotomy was performed above the prosthetic valve plane. We were prepared for a root replacement due to the fact that the TAVI could be merged with the Dacron prosthesis. However, the CoreValve Evolut R 34-mm prosthesis could be carefully explanted with two forceps ( Fig. 1 ). There was some difficulty due to firm integration into the surrounding tissue, but as the tissue was a Dacron graft from the David procedure, there was no concern of damaging the endothelium ( Fig. 2 ). The underlying native valve remnants were bicuspid and heavily calcified. Extensive excision and complete removal of calcifications were necessary to prepare the annulus.

Following annular preparation, a 23-mm Abbott Epic Max bioprosthesis was implanted using individual pledged 2–0 Ethibond sutures. The valve was securely fixed circumferentially, and coronary ostia were confirmed to be patent. The aortotomy was closed with 4–0 Prolene sutures in standard fashion.

After 58 minutes of aortic cross-clamp time, the clamp was removed. Initial ventricular fibrillation occurred, requiring electrical defibrillation. Sinus rhythm was successfully restored. After the reperfusion period, the patient was gradually weaned from CPB under transesophageal echocardiographic guidance, which confirmed good prosthetic valve function without paravalvular leak.

The postoperative course was initially complicated by hemodynamic instability with episodes of bigeminy and ventricular fibrillation, requiring two defibrillations and temporary pacing with a DDD90 mode pacemaker. Notably, the patient had already presented with mild thrombocytopenia preoperatively (platelet count: 128 Gpt/L) in January 2025), which further worsened postoperatively to a nadir of 27 Gpt/L. This raised concern for heparin-induced thrombocytopenia, although anti-PF4 testing was negative.

The patient also developed volume overload and acute kidney injury requiring initiation of dialysis. On postoperative day 3, a right-sided pleural drain was inserted, evacuating 2,800 mL of fluid. Despite prolonged weakness and fluid retention, the patient gradually stabilized with tailored antihypertensive therapy, continued hemodialysis, and control of pleural fluid. He regained alertness and orientation and began mobilization with assistance.

**Fig. 3:**
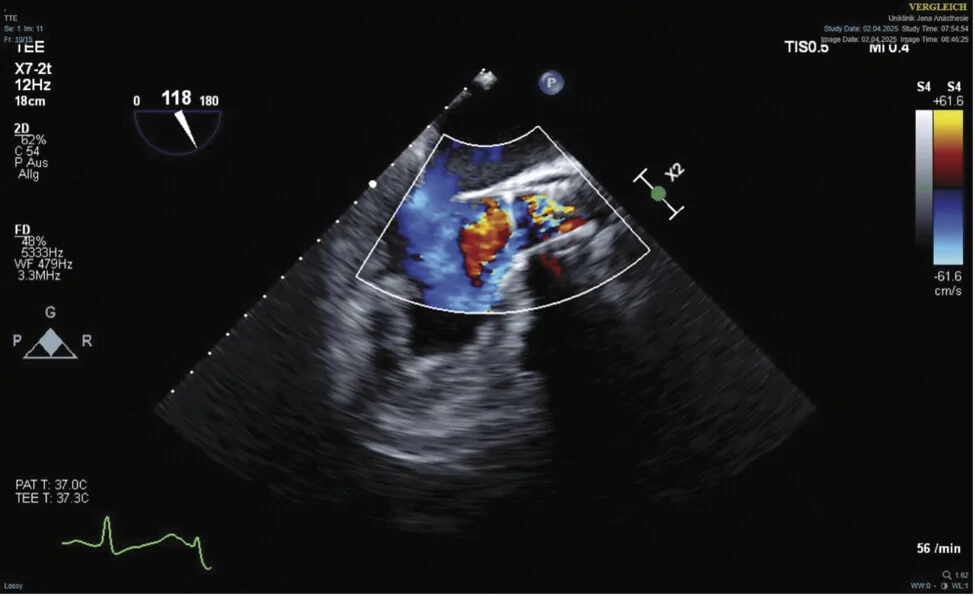
Postoperative echocardiogram showing no signs of paravalvular leakage or any abnormalities.

On postoperative day 15, the patient was discharged to the normal ward. Ultimately, the patient was discharged to outpatient follow-up on postoperative day 22 showing competent bioprothesis in the aortic position ( Fig. 3 ).

## Discussion

This case demonstrates that redo SAVR is feasible and can be life-saving in elderly patients with complex aortic valve histories, including previous valve-sparing root replacement and TAVI.

Current guidelines for aortic valve disease suggest that patients older than 75 years should preferentially undergo TAVI. However, this case report describes a 75-year-old patient who required two interventions in the aortic position. This highlights the need for caution when applying strict age-based cutoffs, emphasizing that clinical decision-making should remain individualized rather than solely guideline-driven. 7 8

It should be acknowledged that the Heart Team decision leading to TAVI implantation in 2018 was made in the context of the available evidence and clinical practice patterns at that time. Although the patient's age of 68 years would have generally favored redo surgery according to the contemporary 2017 guideline recommendations, individualized factors beyond chronological age are routinely incorporated into multidisciplinary decision-making. In particular, the presence of previous complex aortic root surgery following a David procedure may have influenced the perceived procedural complexity and risk profile of redo surgery at that time. This case highlights the importance of individualized Heart Team assessment and reinforces the concept of lifetime management strategies when selecting the initial intervention in younger patients with aortic valve disease.

Beyond the individual clinical course, this case provides important insights for the broader cardiac surgical community, First, it highlights the relative durability of valve-sparing root replacement compared with TAVI, particularly in bicuspid aortic valve anatomy, where long-term outcomes of transcatheter devices remain uncertain. Second, it illustrates that the presence of a synthetic Dacron graft may facilitate safer TAVI explantation by providing a resistant surface, thereby reducing the risk of aortic injury. Finally, it emphasizes the need for careful patient selection and tailored management in reoperations involving prior complex root surgery and transcatheter prostheses.
